# Is there an unmet medical need for improved hearing restoration?

**DOI:** 10.15252/emmm.202215798

**Published:** 2022-07-14

**Authors:** Bettina Julia Wolf, Kathrin Kusch, Victoria Hunniford, Barbara Vona, Robert Kühler, Daniel Keppeler, Nicola Strenzke, Tobias Moser

**Affiliations:** ^1^ Institute for Auditory Neuroscience and InnerEarLab University Medical Center Göttingen Göttingen Germany; ^2^ Auditory Neuroscience and Optogenetics Laboratory German Primate Center Göttingen Germany; ^3^ Auditory Neuroscience & Synaptic Nanophysiology Group Max‐Planck‐Institute for Multidisciplinary Sciences Göttingen Germany; ^4^ Cluster of Excellence "Multiscale Bioimaging: from Molecular Machines to Networks of Excitable Cells" (MBExC) University of Göttingen Göttingen Germany; ^5^ Functional Auditory Genomics Group, Auditory Neuroscience and Optogenetics Laboratory German Primate Center Göttingen Germany; ^6^ Sensory and Motor Neuroscience PhD Program Göttingen Graduate Center for Neurosciences, Biophysics, and Molecular Biosciences Göttingen Germany; ^7^ Institute of Human Genetics University Medical Center Göttingen Göttingen Germany; ^8^ Department of Otolaryngology University Medical Center Göttingen Göttingen Germany; ^9^ Collaborative Research Center 889 University of Göttingen Göttingen Germany

**Keywords:** clinical translation, cochlear implant, gene therapy, hearing impairment, optogenetic hearing restoration, Neuroscience

## Abstract

Hearing impairment, the most prevalent sensory deficit, affects more than 466 million people worldwide (WHO). We presently lack causative treatment for the most common form, sensorineural hearing impairment; hearing aids and cochlear implants (CI) remain the only means of hearing restoration. We engaged with CI users to learn about their expectations and their willingness to collaborate with health care professionals on establishing novel therapies. We summarize upcoming CI innovations, gene therapies, and regenerative approaches and evaluate the chances for clinical translation of these novel strategies. We conclude that there remains an unmet medical need for improving hearing restoration and that we are likely to witness the clinical translation of gene therapy and major CI innovations within this decade.

## Graphical abstract

This review summarizes the clinical translation of gene therapy and cochlear implant innovations and discusses if there is an unmet medical need for improving hearing restoration.

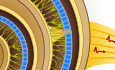

**Glossary**
Adeno‐associated virus (AAV): Single‐strand DNA virus, considered to be non‐disease‐causing, is often used as the vector of choice for expressing transgenes of interest for gene therapeutic approaches. AAVs are engineered to not integrate their DNA into the host genome.
Auditory brainstem response (ABR): Evoked population response reflecting summed synchronized action potentials in the auditory nerve, various auditory brainstem nuclei, and the auditory midbrain.
Channelrhodopsins: Light‐gated ion channels originally found in green algae. When introduced into excitable cells (such as neurons), channelrhodopsins enable precisely controlled light‐induced action potential generation.
Cochlear implant (CI): Neuroprosthetic device, which directly stimulates the auditory nerve and thereby partially restores hearing in patients suffering from profound sensorineural hearing loss.
Cochlear optogenetics (in this review): Optogenetic stimulation of spiral ganglion neurons.
Electrical cochlear implant (eCI): CI that stimulates the auditory nerve by electrical impulses.
Gene correction (=Gene editing): A gene therapeutic approach correcting the DNA sequence of a dysfunctional allele with detrimental effects to gain expression of a gene product with normal function.
Gene replacement: A gene therapeutic approach adding a functional gene copy to replace the present nonfunctional allele.
Gene supplementation: A gene therapeutic approach to augment the expression of a functional allele.
Hereditary hearing loss: Hearing loss caused by mutations in genes involved in the normal function of the ear and is hereditable. Discrimination of syndromic (affecting more organs than the ear) or nonsyndromic forms (affecting only hearing) that are further specified by hereditability and gene affected as autosomal dominant (DFNA, 80 types), autosomal recessive (DFNB, 117 types), X‐linked (DFNX, 6 types) or mitochondrial.
Infrared light: Electromagnetic irradiation with a wavelength longer than visible light (700 nm to 1 mm wavelength).
Intraneural inserted eCI: An array with slanted needle electrodes penetrates the auditory nerve at the internal auditory canal.
Light‐emitting diode (LED): Semiconductor light source, which emits photons when the electric current is applied.
Mechanotransduction: Conversion of mechanical stimulation such as pressure waves into electric signals by mechanosensory hair cells.
Modiolus: Central axis of the cochlea housing the spiral ganglion.
Optogenetics: Genetic modification of biological tissue enabling control of cells by light.
Optical Cochlear Implant oCI: Active vs. passive implementation, while “active” oCIs integrate optoelectronics such as micro‐LEDs generating light directly inside the cochlea, “passive” oCIs employ light‐guiding waveguide arrays to pipe the light from emitters placed in the extracochlear titanium‐housed stimulator into the cochlea.
Organ of Corti: Sensory organ of the inner ear, housing inner and outer hair cells, and various supporting cells.
Ossicles: Three bones (malleus, incus, and stapes) in the middle ear, which amplify and relay pressure waves from the outer ear arriving at the eardrum to the inner ear via the oval window.
Otoferlin (protein), *OTOF* (gene): A transmembrane protein expressed in inner hair cells essential for hair cell synaptic transmission, mutations in the *OTOF* gene coding for otoferlin cause autosomal‐recessive hearing loss DFNB9.
Photocurrents: Ionic currents mediated by light‐gated ion channels upon illumination.
Phototoxicity: Damage of cells or tissue evoked by intense exposure to light.
Ribbon synapses: Specialized synapses in the inner ear and retina, which are characterized by electron‐dense structures (ribbons) that tether synaptic vesicles to presynaptic active zones.
Rosenthal's canal: Cavity in the modiolus housing the cell bodies of spiral ganglion neurons.
Scala tympani: Perilymph‐filled intracochlear cavity extending from the round window to the helicotrema.
Sensorineural hearing loss: Hearing loss resulting from dysfunction of the cochlea and/or spiral ganglion.
Spectral selectivity: Precision by which the cochlea can encode sound frequency upon acoustic, electrical, or optogenetic stimulation.
Spiral ganglion neurons (SGNs): Bipolar neurons housed in Rosenthal's canal in the modiolus who innervate hair cells and whose axons form the auditory nerve, projecting to the cochlear nucleus in the auditory brainstem.
Transmembrane channel‐like 1 protein (TMC1): A protein expressed in inner hair cells essential for mechanotransduction. Mutations in TMC1 are causative for autosomal‐dominant (DFNA36) or autosomal‐recessive (DFNB11) hearing loss.
Tonotopy: Place‐frequency code in the auditory system.
Transduction: Gene transfer by viral vectors.
Waveguide array: Array of physical structures which guide electromagnetic waves in the optical spectrum to their target structures.

## Introduction—Hearing, hearing impairment, and hearing restoration

### Physiological hearing

Acoustic signals or sound, including human speech and music, are air pressure waves of different frequencies and amplitudes that fluctuate in time. Once picked up by the outer ear, pressure waves vibrate the ear drum, which along with the attached chain of ossicles aligns the low impedance of the air and the high impedance of fluids in the cochlea where sound is transformed into nerve signals (Kandel *et al*, 2012). The cochlea then decomposes the frequency components of the signal‐forming pressure waves on the basilar membrane based on the graded intrinsic mechanical properties of the membrane (Kandel *et al*, 2012). Specifically, high frequencies most effectively vibrate the membrane at the cochlear base where it is narrow and stiff, while low‐frequency waves vibrate maximally the softest and widest area of the membrane at the cochlear apex. This way, this micromechanical spectral analyzer establishes a frequency map in the cochlea (also known as the tonotopic axis; Kandel *et al*, 2012) that is read out by mechanosensory inner and outer hair cells (IHCs and OHCs) (Fettiplace, 2017; Effertz *et al*, 2020).

**Figure 1:**
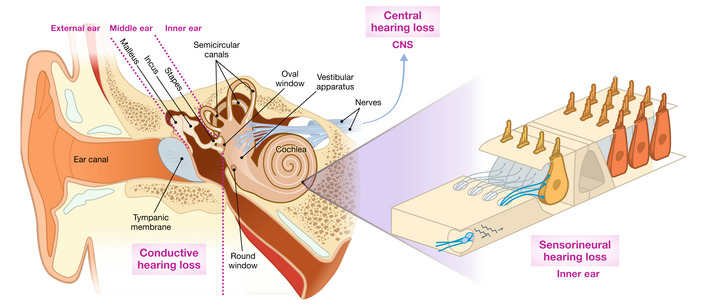
Normal hearing and hearing impairment Schematic illustration of the auditory periphery. Soundwaves are funneled by the pinna of the outer ear into the ear canal, vibrate the tympanic membrane, which is connected via three small ossicles (malleus, incus, and stapes) to the oval window of the snail‐shaped cochlea. The cochlea is organized in a tonotopic manner, meaning low frequencies are represented in the apical turn and high frequencies towards the base. Sound encoding takes place in spiral ganglion neurons (SGNs) that are driven by glutamatergic transmission at ribbon synapses with inner hair cells (IHCs), which are part of the organ of Corti (inset). IHCs are each innervated by ~ 5–30 SGNs, which are located at some distance to the hair cells in the Rosenthal's canal forming the auditory nerve heading towards the brain. Three rows of outer hair cells (OHC) provide cochlear amplification and compression. Hearing impairment caused by alterations of outer and/or middle ear is defined as conductive hearing loss, while sensorineural hearing loss describes dysfunctional or missing hair cells. Lesions of the central auditory system cause central hearing loss.

IHCs form one and OHCs three rows in the organ of Corti running along the entire length of the basilar membrane. Both carry hair bundles at their apex as the mechanoelectrical transduction machinery: Deflection of the bundle opens mechanotransducer channels, which enables depolarizing cation—mostly potassium—influx into the hair cell (Hudspeth, 2014; Fettiplace, 2017; Effertz *et al*, 2020). Hair bundles of OHCs are mechanically connected to the tectorial membrane that lies above the organ of Corti. The traveling wave causes movement of the organ of Corti, which sits on the basilar membrane, relative to the tectorial membrane. This directly deflects the hair bundles of OHCs and indirectly, likely by fluid flux, the hair bundles of IHCs. The amplitude of the traveling wave determines the extent of IHC and OHC activation at the respective cochlear location and the spatial spread of excitation, that is, hair cell activation, on the frequency map (Fig 1; Békésy & Wever, 1960; Chatterjee & Zwislocki, 1998). This tonotopic organization is kept throughout the auditory pathway up to the cortex.

OHCs employ electromotility to feed mechanical energy into the vibration of the basilar membrane and thereby amplify the traveling wave for soft sounds (Ashmore, 2008; Hudspeth, 2014; Fettiplace, 2017). IHCs in turn employ sophisticated ribbon synapses (Matthews & Fuchs, 2010; Safieddine *et al*, 2012; Wichmann & Moser, 2015; Moser *et al*, 2019) to transmit the sound information to the spiral ganglion neurons (SGNs), which generate the auditory code (Geisler, 1998; Heil & Peterson, 2015). The spike rate and the number of activated SGNs encode sound intensity and the location of SGN activation along the cochlea encodes the frequency. Sub‐kHz frequencies are additionally encoded by the time of spiking because the sub‐millisecond precision of cochlear processing allows SGNs to spike in a fixed relation to the stimulus cycle (Geisler, 1998; Heil & Peterson, 2015).

The afferent auditory pathway processes via highly specialized neurons and networks. For instance, the cochlear nucleus, the first stage of the central pathway, receives converging and diverging input of SGNs to process the information on stimulus timing, frequency, and intensity. Bushy cells of the cochlear nucleus use coincidence detection to further sharpen the temporal code represented in the SGNs and pass it on to the neurons of the olivary complex (Oertel, 1999). These use precisely timed excitation and inhibition for calculating the localization of the sound source in the horizontal plane from the input of both ears (Grothe *et al*, 2010). The various neural networks of the auditory brainstem project to the inferior colliculus, the auditory midbrain, that integrates the information and projects to the primary auditory cortex via the thalamic medial geniculate (Kandel *et al*, 2012). The formation of “auditory objects,” such as a piece of music, human speech, or a barking dog, is thought to start in the midbrain. Auditory information is eventually integrated with other sensory information in associative cortices to represent the objects we perceive and act on them (Kandel *et al*, 2012).

### Hearing impairment

Hearing impairment is the most prevalent sensory deficit and has major socioeconomic impacts. According to the World Health Organization (WHO), 466 million people (432 million adults and 34 million children) suffer from disabling hearing loss (HL), and more than 700 million people are estimated to be affected by disabling hearing loss in 2050 (WHO, 2021). Disabling hearing impairment refers to an increase in the pure tone threshold of greater than 40 dB in the better hearing ear in adults and 30 dB in children. 1–2 out of 1,000 babies and another 1 in 1,000 children are affected until adolescence (Morton & Nance, 2006). The prevalence increases with age: Approximately one‐third of people suffering from the disabled hearing are over the age of 65 (WHO, 2021). If left untreated, hearing impairment diminishes an individual's ability to communicate with others. Normal development of vocal speech in children requires good hearing and even mild hearing impairment therefore affects vocal speech acquisition. Hearing impairment in adults affects their private and professional activities, and often leads to social isolation, thereby increasing the risk of depression and, in the elderly, of cognitive decline (Livingston *et al*, 2017; Montero‐Odasso *et al*, 2020). WHO estimates that unaddressed hearing loss poses an annual global cost of US$ 980 billion (WHO, 2021). This includes health sector costs (excluding the cost of hearing devices), costs of educational support, loss of productivity, and societal cost.

The pathophysiology of hearing impairment can be classified according to the site of the lesion (Fig 1; Eggermont, 2017a). Conductive hearing impairment arises from disturbed sound conduction in the outer and/or middle ear. Central hearing impairment is caused by lesions of the central auditory pathway. The most common form of hearing impairment is sensorineural hearing loss, where IHCs, OHCs, and/or SGNs and/or other cell types in the cochlea are dysfunctional or lost (Eggermont, 2017a; Moser *et al*, 2013) owing to genetic defects or, more commonly, a variety of external factors such as noxious sounds, treatment with ototoxic drugs such as some antibiotics (e.g., aminoglycosides), anti‐cancer drugs (e.g., cisplatin), and others (Eggermont, 2017b).

More than half of the cases of congenital sensorineural hearing impairment are attributed to single‐gene defects (Morton & Nance, 2006). Such monogenic hearing impairment can be nonsyndromic or syndromic with autosomal‐recessive inheritance as the most common form. These “deafness” genes encode a broad range of proteins: transcription factors, extracellular matrix proteins, ion channels, and pumps, or multidomain proteins of various functions that assume essential roles in IHCs, OHCs, SGNs, and/or other cochlear cell types (Dror & Avraham, 2010).

Defects impairing mechanoelectrical transduction, that is, the mechanosensitive influx of potassium into IHCs and OHCs, globally disrupt cochlear function. This can happen directly by mutations affecting mechanoelectrical transduction, or indirectly, by defects disrupting the stria vascularis that powers the cochlea or those altering the cochlear potassium cycle that returns potassium from hair cells to the stria vascularis. Mutations selectively disrupting OHCs reduce acoustic sensitivity and frequency selectivity. Impairment of the afferent synapse between IHCs and SGNs disrupts synaptic sound encoding (auditory synaptopathy, (Moser & Starr, 2016)). Initially, deafness genes and their mutations were mainly identified by linkage analysis with subsequent Sanger sequencing of candidate genes within the linked region (Petit *et al*, 2001; Duman & Tekin, 2012). Nowadays, high‐throughput sequencing with next‐generation sequencing (Shearer & Smith, 2015) and subsequent bioinformatic analysis has become a standard procedure.

Complex genetic hearing impairment includes noise‐induced and age‐related hearing loss (Bowl & Dawson, 2019). Noise‐induced hearing loss is the leading occupational disorder. Generally, excessive exposure to loud sounds, occupationally or in private life, is an increasing threat to healthy hearing. The louder the noise, the shorter an exposure is sufficient to cause the same amount of damage. Long and strong noise exposure [greater than 85 dB(A) for 8 h a day for years, see statements of OHSA and NIOSH in the useful links section] can increase in auditory threshold due to loss of OHCs but can also affect IHCs. However recently, it has been observed that noise‐induced, probably excitotoxic, damage to the afferent synapses of IHCs (auditory synaptopathy) is present even before a permanent increase in auditory threshold commences, hence the term “hidden hearing loss” (Liberman, 2017).

Otolaryngology provides excellent solutions to treat conductive hearing loss by microsurgery of the middle ear or prostheses and implantable hearing aids. Yet, as of today, we lack causative treatment options for sensorineural hearing loss. The state of the art of hearing restoration is hearing aids for mild and moderate threshold increases and electrical cochlear implants when acoustic amplification is no longer sufficient. This review focuses on current and future treatment options for profound hearing impairment and deafness, that is, cases that currently would be best served with cochlear implants. Future approaches for hearing restoration will likely include pharmacology, gene therapy, optogenetics, and regenerative medicine.

## Cochlear implants enable bionic hearing

**Figure 2:**
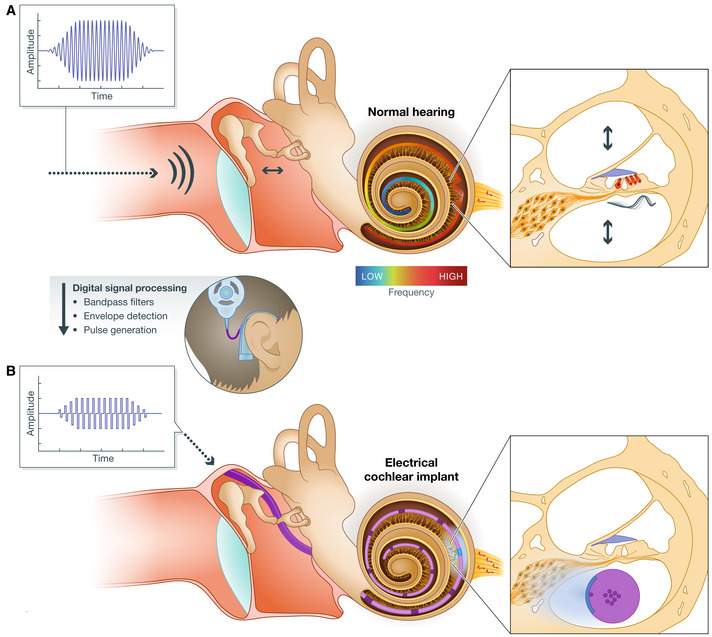
Normal and electrical hearing (A) Acoustic hearing: Sound pressure waves in the air travel along the ear canal and are relayed via the ossicles into the intracochlear fluid, where they are decomposed in a frequency‐dependent manner (center). A traveling pressure wave along the basilar membrane activates mechanosensitive hair cells (red) in the organ of Corti at the respective cochlear location and thereby starts the information flow in the auditory system via synaptic transmission from IHCs to SGNs (right). The precise frequency mapping (tonotopy) is visualized through the basilar membrane (see color bar). (B) Electrical hearing: Acoustic signals are analyzed by an external processor, which extracts predominant frequencies and corresponding amplitudes of the signal. The extracted frequencies are mapped to distinct stimulation sites, so that SGNs around the tonotopic region that would be activated by hair cells for a given sound frequency in physiological hearing (A) are then directly activated by the implanted electrodes (B).

Introduced in the 1970s following a collaboration between visionary engineers, otolaryngologists, and courageous patients, the electric cochlear implant (eCI) is arguably the most successful neuroprosthesis, and it is currently used by more than a million people. The eCI consists of an external component—a microphone(s), an audioprocessor, and a battery pack—and an internal part with an electrical pulse generator and a linear electrode array that is placed into the fluid‐filled cochlear compartment (scala tympani) along the tonotopic axis (Fig 2). External and internal components are inductively connected by two magnetically coupled coils, to power the implant and transmit information. The processor extracts predominant frequency components from the surrounding auditory environment and maps them to the eCI electrodes located at different positions along the tonotopic axis. The eCI electrically stimulates SGNs around these electrodes taking advantage of the intrinsic place‐frequency code of the cochlea with pulse amplitudes mapped to sound intensity in a given frequency band. By direct SGN stimulation, the eCI bypasses dysfunctional or lost IHCs. Despite the use of only 12–24 electrodes representing fixed frequency bands, eCIs enable open speech comprehension in most users.

Since the introduction of the multichannel eCI, its performance has been enhanced in several ways, including advances in coding strategies (the software that analyzes the sound and operates the internal part); increasing the stimulation rates of the implant (~ 1 kHz per electrode); and employing wireless connectivity (Lenarz, 2017; Zeng, 2017). However, meaningful use of the implant typically requires practice with “electrical hearing” over a rehabilitation period of 6–12 months. Not only the time required for reaching speech comprehension varies but so does the eventual outcome of eCI rehabilitation. The outcome of eCI rehabilitation generally depends on several parameters that include the number and functional status of SGNs (e.g., Starr *et al*, 2008; Cosetti & Waltzman, 2012), the coverage of the tonotopic axis by the electrical array, the physical trauma associated with the surgery, and the cognitive capabilities of the CI user and efforts undertaken in rehabilitation (e.g., Lenarz, 2017). For children with hearing impairment, the sooner eCIs are provided the better the outcome in terms of hearing, speech, and mental development in particular (Sharma *et al*, 2020).

## Limitations of current cochlear implants and unmet medical need for improved hearing restoration

We conducted a survey among adult patients from the Department of Otolaryngology of University Medical Center Göttingen who were actively using a cochlear implant for 6 months or more during the time of the survey period, about their long‐term experience with the implant. This custom questionnaire specifically addressed eCI users about their perspectives on future means of hearing restoration in conjunction with the commonly used questionnaires SSQ‐12 (Speech, Spatial and Qualities of Hearing scale suitable for clinical use; Gatehouse & Noble, 2004, 12) and IOI‐HA (International Outcome Inventory for Hearing Aids; Cox *et al*, 2000). Of the 79 eCI users who completed the custom questionnaire, 68% were older than 60 years; 72% had unilateral implantation; and the median number of years participants had their implant was 3, from 6 months to 25.5 years.

**Figure 3:**
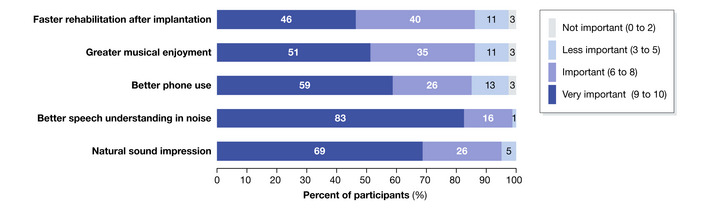
Patient perspective on the importance of improved hearing restoration Result of a survey conducted among 79 eCI patients with eCI. Participants ranked the importance of the hearing experience from 0 (not important) up to 10 (very important), and responses were separated into 4 categories of importance: not important (0 to 2), less important (3 to 5), important (6 to 8), and very important (9 to 10). The majority of respondents rated hearing of natural sounds, speech, phone calls, and music as quite important. In addition, fast rehabilitation after implantation seems relevant to users.

Overall, the respondents expressed a need for improving the performance beyond that experienced with their current eCI (Fig 3). In particular, they identified three key limitations: difficulties with understanding speech in situations with competing background noise or multiple speakers; unnatural auditory perception; and limited music experience. In addition, they strongly desired faster hearing rehabilitation after CI surgery. Physiologically, the main cause for the limited performance of eCI hearing is the wide current spread of electrodes in the salty fluid space of the cochlea. An electrical current delivered from any electrode recruits large populations of tonotopically different SGNs, limiting the precision by which eCIs can utilize the intrinsic place‐frequency code of the cochlea (Shannon, 1983; Kral *et al*, 1998) and the coding of sound intensity (Zeng, 2004; Miller *et al*, 2006).

## Future strategies for improved hearing restoration

### Innovations of cochlear implants

Clinically available innovations include but are not limited to wireless connectivity, directional hearing using microphone arrays (contralateral hearing aid or cochlear implant), and advanced sound preprocessing that will eventually involve AI‐powered analysis of acoustic scenes. Another major development has been the totally implantable cochlear implant (TICI; Mi2000, ClinicalTrials.gov Identifier: NCT04571333). In terms of improving the transmission of time and frequency information, there have been recent advancements in temporal fine structure coding for electrodes that carry low‐frequency information (Dhanasingh & Hochmair, 2021) and multipolar stimulation for improved frequency selectivity (Bierer, 2010).

In this review, we will focus on three recent developments that combine advances in medical device engineering with biotechnology to genetically modify target cells: improvement of the electrode‐neural interface, using light for the stimulation of the auditory nerve, and hybrid optical‐electrical stimulation.

For improving the electrode‐neural interface, two major strategies are being pursued: electrode arrays directly penetrating the auditory nerve and attracting SGN neurites towards the electrodes of eCI placed intrascalar (the current state‐of‐the‐art placement in scala tympani).

Pioneered by Middlebrooks and Snyder (2007), intraneurally inserted electrode arrays have been shown to improve the spectral selectivity in preclinical studies using silicon shaft multielectrode arrays. Moreover, the required current is lower than for intrascalar eCIs. Currently, the approach is being prepared for a clinical trial by the Lenarz and Lim teams in collaboration with Blackrock and MED‐EL companies (Lenarz & Lim ARO Midwinter Meeting 2020 & Conference on Implantable Auditory Prosthesis 2021, https://youtu.be/eZJPqRmAzSA). They plan to use a Utah array with slanted needle electrodes for penetrating the auditory nerve. Challenges of the approach include: a more complicated mapping of frequencies to electrodes than for intrascalar eCI, the risk of scar formation increasing the electrode resistance, and surgical access to the implantation site.

Attracting SGN neurites closer to the intrascalar electrodes is a longstanding concept and includes efforts such as a combination of long‐term electrical stimulation and administration of neurotrophic factors GDNF or BDNF (review in Pettingill *et al*, 2007), and transgenic expression of neurotrophic factors (e.g., BDNF) in the mesenchyme of the scala tympani (Pinyon *et al*, 2014). The hypothesis is that close proximity or even direct contact of neurites to the electrodes will lower the current thresholds for a given electrode and thereby more selectively recruit those proximal neurons over the other SGNs.

Using light as an alternative for bionic sound encoding promises to overcome the major bottleneck of eCIs: poor spectral selectivity. As light can be better confined in space, it enables SGN stimulation with higher spatial selectivity, resulting in improved spectral selectivity (Fig 5 bottom; Izzo *et al*, 2007; Richter *et al*, 2011; Hernandez *et al*, 2014; Jeschke & Moser, 2015; Moser, 2015). Richter and colleagues have used pulsed infrared lasers to stimulate SGNs (Izzo *et al*, 2007). The energy threshold for neural activation was reported to be at least 15 μJ per pulse (Izzo *et al*, 2007; Tan *et al*, 2015). A proof‐of‐concept clinical study is planned to start in early 2022 (ClinicalTrials.gov Identifier: NCT05110183). The method has remained somewhat controversial; however, the feasibility of direct infrared stimulation of SGNs has been challenged by studies in other laboratories (Teudt *et al*, 2011; Thompson *et al*, 2015; Kallweit *et al*, 2016; Baumhoff *et al*, 2019).

**Figure 4:**
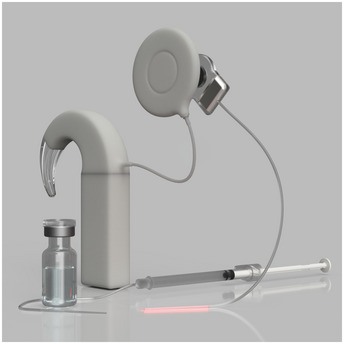
Optogenetic hearing restoration will likely build on combining AAV‐mediated gene therapy for ChR expressing in SGNs with waveguide‐based oCI for spectrally selective SGN stimulation Illustration of a future oCI consisting of a red multibeam waveguide, a sound processor with a newly developed sound coding strategy, which enables complete exploitation of the optical stimulation. In front a catheter and a vial carrying the AAV symbolizing the need for gene therapy.

One alternative to infrared stimulation of the auditory nerve is using optogenetics. It has lower light requirements and offers a molecularly defined and therefore tunable mechanism of neural activation for more selective stimulation of SGNs. About two decades ago, light‐gated ion channels, so‐called Channelrhodopsins (ChRs; a subtype of microbial opsins), were demonstrated to mediate light‐driven action potentials in mammalian neurons (Nagel *et al*, 2003; Boyden *et al*, 2005). This has made optical cochlear implants (oCI) feasible by way of genetically rendering SGNs light‐sensitive (Hernandez *et al*, 2014). As such, optogenetic hearing restoration requires the combination of gene therapy and the oCI as a medical device (Fig 4).

Gene therapy targeting SGNs for transgenic expression of ChRs will likely employ local administration of nonpathogenic adeno‐associated viruses (AAVs) to the cochlea. Current preclinical work tries to identify the most suitable combination of ChR, AAV, and promoter with good tropism for SGNs and includes AAV2/6, AAV2/9, AAV‐PHP.B, and AAV‐PHP.eB (Hernandez *et al*, 2014; Keppeler *et al*, 2018; Mager *et al*, 2018; Bali *et al*, 2021; Huet *et al*, 2021). Provided administration of AAV can be restricted to the cochlea, broad‐acting, but efficient neuronal promoters, such as the human synapsin promoter, can selectively express ChRs in SGNs (Wrobel *et al*, 2018). Transduction efficiency is governed by the type and number of AAV particles, the strength of the promoter and the accessibility and susceptibility of the target cells for the viral vector, which, again, is determined by the route of administration.

The ideal ChR for optogenetic hearing restoration would combine large photocurrents, red‐light activation, and fast‐closing kinetics. Large photocurrents are required for keeping the energy budget of the oCI to an acceptable level and can be achieved by high ChR density in the plasma membrane and by large ChR conductance. ChR generally has low single‐channel conductance (e.g., 40 fS for ChR2; Feldbauer *et al*, 2009); the oCI would therefore require a high ChR density within SGNs, which increases the risk for proteostatic stress and detection by the immune system. Recently, ChRs with larger conductance have been reported that promise greater light sensitivity (Marshel *et al*, 2019; Hososhima *et al*, 2020; Kishi *et al*, 2022). Activation by low‐energy, long‐wavelength photons reduces light scattering in the tissue to improve spatial selectivity along with reducing the risk of phototoxicity. Finally, fast‐closing ChR kinetics after light‐off is required for achieving SGN firing rates in the range of a few hundred Hz and sub‐millisecond precision of spike timing (Heil & Peterson, 2015). Naturally occurring fast‐closing ChRs such as Chronos (Klapoetke *et al*, 2014; Duarte *et al*, 2018; Keppeler *et al*, 2018) and site‐directed mutagenesis of previously identified ChRs that generate fast Chrimson (Klapoetke *et al*, 2014) variants f‐Chrimson and vf‐Chrimson (Mager *et al*, 2018; Bali *et al*, 2021) could serve this requirement. Shortening the lifetime of the ChR open state, however, reduces the charge transfer per absorbed photon, thereby increasing the light requirement to generate a spike. Overall, there is a trade‐off between temporal precision and the needed energy for optogenetic SGN stimulation.

**Figure 5:**
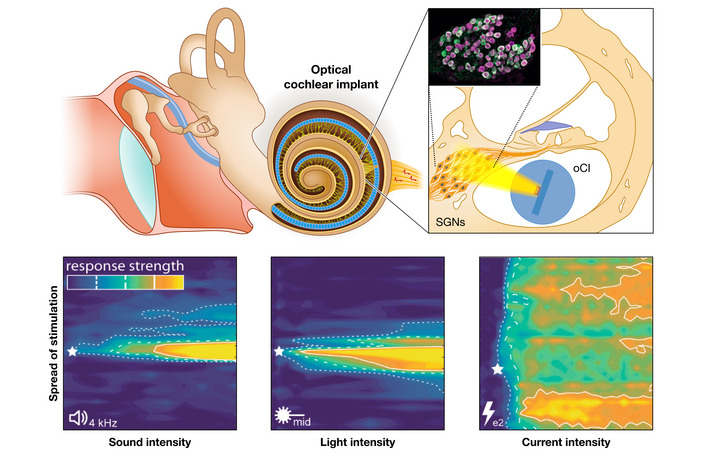
Principle of optogenetic hearing restoration Top: An emitter array is placed in scala tympani and provides spatially confined optical stimulation of ChR expressing SGNs (inset shows immunofluorescently marked ChR (green) expressing SGNs, identified by context marker parvalbumin (magenta) in rodents). Bottom: demonstration of near physiological spectral selectivity. Confined or spectral selective midbrain activity for optogenetic (middle panel), acoustic (left), but not for electrical (right) stimulation with poor spectral selectivity.

Development of the medical device focuses on adapting existing CI components—speech processor, transmission coil and magnet, titanium housing, electrical feedthrough, and potentially, the implanted electronics—for optical stimulation. While “active” oCIs integrate optoelectronics such as micro‐light‐emitting diodes (μLED) which generate light inside the cochlea, “passive” oCIs employ light‐conducting waveguide arrays to pipe the light from emitters in the extracochlear titanium‐housed stimulator into the cochlea (Fig 5). Passive oCIs require optical feedthrough from the titanium‐housed stimulator towards the waveguide array, which risks light losses at the in‐ and outcoupling sites but promises better long‐term stability and less heat generation inside the cochlea. Active implants minimize coupling losses of light but require hermetic yet mechanically flexible and optically transparent encapsulation. Regardless of active or passive oCI implementation, optical coding strategies need to be developed that accommodate more stimulation channels and longer effective stimulation pulses than used in eCI.

We and others have demonstrated safe and stable AAV‐mediated ChR expression in SGNs (Fig 5) in rodents (mice, rats, and gerbils) upon intracochlear application of a single AAV dose (Keppeler *et al*, 2018; Huet *et al*, 2021; Bali *et al*, 2022). Optogenetic stimulation of the auditory nerve activates the auditory pathway up to the cortex and elicits behavioral percepts that are generalized to auditory perception (Wrobel *et al*, 2018; Keppeler *et al*, 2020). Nearly physiological spectral selectivity, fundamentally exceeding that of state‐of‐the‐art eCI, could be demonstrated for optogenetic stimulation *in vivo* and *in silico* (Dieter *et al*, 2019, 2020; Keppeler *et al*, 2020; Fig 5). Preclinical multichannel oCI systems based on blue‐light‐emitting μLED restored hearing in rodent models of human deafness (Keppeler *et al*, 2020).

First applications of optogenetic hearing restorations in humans are planned for late 2026 (Fig 4). The approach will first entail intracochlear administration of a gene therapy medicinal product based on an AAV‐construct carrying a neuron‐specific promotor and a suitable ChR candidate. Preclinical studies indicate two alternative approaches: A catheter‐based application into scala tympani via the round window (Thirumalai, *unpublished*); or an injection into the modiolar axis via the apical turn of the cochlea (Wrobel *et al*, 2021). Both procedures can likely be performed through the ear canal in general or local anesthesia. A few weeks later, prior to oCI implantation, successful optogenetic stimulation can be probed by measurements of optically evoked stapedial reflexes, SGN compound action potentials, and/or auditory brainstem responses using a laser‐coupled optical fiber.

Combining optical and electrical stimulation of SGNs could use the advantages of either technique. For example, the combination of electrical and infrared stimulation of the auditory nerve in deaf white cats reduced the required radiant (light) energy (Richter *et al*, 2014). Another study combining electrical and optogenetic stimulation in mice expressing a ChR2 variant showed that sub‐threshold optical stimulation was able to lower the threshold for subsequent electrical stimulation (Richardson *et al*, 2021).

### Gene therapy of the cochlea

Monogenic hearing impairment is an attractive target for gene therapy if cochlear development proceeds normally and if its structure is preserved in the neonatal cochlea despite disrupted function. Genetic diagnostics and preclinical work in animal models promise that hearing restoration by gene therapy could become available within the coming decade (Kleinlogel *et al*, 2020). However, this approach will only be an option for those gene defects that do not majorly alter the cochlear structure and would only be available for a small patient population. This can hamper clinical translation and makes these future therapies costly. For example, three biotech‐driven projects to develop gene therapy for the *OTOF* gene mutant in auditory synaptopathy DFNB9, one of the most popular targets, seem to compete for a quite limited number of cases in Europe and the USA. While no public information on planned pricing is available, costs are likely to be substantial as in other gene therapies. For example, a single dose of Luxturna—a treatment for Leber's congenital amaurosis type 2 (LCA2), and the first FDA‐approved gene therapy—to treat one eye was initially priced at US$800,000.

Inner‐ear gene therapy approaches target hair cells, SGNs, or other cellular populations such as supporting cells. Dependent on the causative molecular and biological mechanisms, different strategies for gene therapy are being explored for restoring normal gene expression (reviewed in Delmaghani & El‐Amraoui, 2020; Kleinlogel *et al*, 2020). Gene replacement or gene supplementation approaches replace nonfunctional alleles with a functional allele or supplement (augment) the expression of the functional allele. Gene correction approaches typically suppress or edit dominant malfunctional alleles. Examples include gene replacement in *OTOF*‐related auditory synaptopathy DFNB9 and gene correction for editing of *TMC1* (review in Ahmed *et al*, 2017; Kleinlogel *et al*, 2020). Alternative gene therapy approaches follow transgenic strategies for expression of opsins for optogenetic hearing restoration, for neurotrophic repair of IHC synapses or for HATH‐1 driven trans‐differentiation of supporting cells to generate hair cells [ClinicalTrials.gov identifier: NCT02132130]. The latter trial demonstrated a favorable safety profile of adenovirus‐mediated inner‐ear gene therapy, but its efficacy was limited if present at all. Efforts to drive supporting cell trans‐differentiation into hair cells by inhibiting Notch signaling via Gamma‐secretase inhibitors have recently entered clinical trial [EudraCT: 2016–004544‐10, ClinicalTrials.gov Identifier: NCT05061758].

Most preclinical gene therapies and ongoing or planned clinical studies employ nonpathogenic AAVs that are less immunogenic than adenoviruses. Sendai virus, Vaccinia virus, and Herpes simplex virus have been evaluated preclinically for use in the inner ear but were not chosen for clinical trials possibly due to modest transduction rates (Kleinlogel *et al*, 2020). Although AAVs have been successfully applied in gene therapy of the eye, such as in the case of Luxturna, delivery of the coding sequence of some large genes, such as *OTOF* (~ 6 kb), is not straightforward owing to the limited packaging capacity of standard AAV (< 4.7 kb; Grieger & Samulski, 2005). This packaging problem has been tackled in preclinical gene replacement trials in DFNB9 mouse models using two approaches: dual‐AAV—that is two different AAVs that carry 5′ and 3′ fragments of the coding sequence that recombine inside the IHC (Akil *et al*, 2019; Al‐Moyed, 2019)—and AAV overload: forcing the entire sequence into one AAV (Rankovic *et al*, 2021). Hearing of the deaf DFNB9 mouse models could be partially restored as shown by auditory brain stem recording (Akil *et al*, 2019; Al‐Moyed, 2019; Rankovic *et al*, 2021) and behavioral evaluation (Rankovic *et al*, 2021). Recessive loss of gene function (DFNBX) and dominant haploinsufficiency (e.g., DFNA36) are amenable to gene replacement, supplementation, and correction. Aside from DFNB9, current efforts target Usher Syndrome Type 3A (defects of *CLRN1* coding for clarin‐1), DFNB8 (defects of *TMRPSS3* coding for Transmembrane protease serine 3), and DFNB16 (defects of *STRC* coding for stereocilin).

Dominant‐negative alleles can only be tackled by gene correction. Gene therapy targeting *TMC1*, coding for the candidate mechanotransducer channel of hair cells, has been intensely studied in mice for genome editing by CRISPR‐Cas9 and base editing (Askew *et al*, 2015; Gao *et al*, 2018; György *et al*, 2019; Yeh *et al*, 2020; Zheng *et al*, 2022). In the eye, a first phase I and II clinical gene correction trial, building on prior studies on ocular gene therapy, [BRILLIANCE, ClinicalTrials.gov Identifier: NCT03872479] (Maeder *et al*, 2019) is planned to test the safety and feasibility of Cas9‐mediated repair of the most common cause of inherited childhood blindness (Leber's congenital amaurosis 10).

## Regenerative approaches

Hair cells are the primary target for regenerative approaches to hearing restoration. Building on pioneering work (summarized in Brigande & Heller, 2009), the field has moved forward in devising protocols for generating hair cells, SGNs, or even inner‐ear organoids from stem cells (recent reviews Janesick & Heller, 2019; Takeda *et al*, 2018; Wang & Puel, 2018; Sekiya & Holley, 2021). Koehler *et al* (2017) developed a protocol for generating inner‐ear organoids from human‐induced pluripotent stem cells (iPSCs) (Koehler *et al*, 2017). Since then, several protocols for generating inner‐ear cells from human iPSCs have been developed (for review see ref. Tang *et al*, 2020), which may contribute to a more efficient generation of otic organoids. Ideally, human hair cells or SGNs could be transplanted into the cochlea to replace the degenerated cells. However, hair cells or SGNs are quite sensitive and dissociation and transplantation remain challenging. Early attempts of embryonic stem cell injections into the inner ear have not been straightforward, as there is a low probability of producing otic cells and a high risk for potential teratoma formations (Chen *et al*, 2018). Transplanting otic progenitor cells (OPCs) has advantages, too. A few studies have demonstrated grafted cells with characteristics similar to HCs and SGNs (Chen *et al*, 2018; Lopez‐Juarez *et al*, 2019), while one study demonstrated partial hearing restoration (Chen *et al*, 2012).

The remaining challenges for clinical translation of regenerative approaches include organized growth throughout the bony housing of Rosenthal's canal and appropriate synaptic reconnection to the cochlear nucleus for signal transmission to higher brain regions. Further strategies include tissue engineering through scaffolds to enable guided neurite growth (Hackelberg *et al*, 2017), the aforementioned neurotrophin‐based stimulation, and small molecules targeting glial cells for reprogramming into SGNs (Chen *et al*, 2012).

Although regenerative medicine has been seen as an attractive form of therapy for several decades, its overall success in clinical translation is poor (doi: 10.1111/cts.12736). According to allied market research, the market for regenerative medicine would be worth US$67.5 billion by 2020 (doi: 10.1002/cpt.549). Notwithstanding this optimistic projection, to our knowlegde, only one regenerative medicine therapy has achieved regulatory approval (hematopoietic stem cell transplant for blood disorders and other immunodeficiencies [doi: 10.5966/sctm.2015‐0275]). Although the number of clinical trials for regenerative medicine therapies continues to increase, the field remains largely experimental—evidence on safety and efficacy is still lacking, and we have yet to see a “gold standard” regenerative medicine therapy for clinical application (https://doi.org/10.2217/rme.13.80).

## Conclusion and outlook

The WHO forecasts a dramatic increase in patients with disabling hearing impairment in the coming decades. Current treatments improve the lives of many patients, but the sensory experience remains less than optimal. Thus, it is essential to develop novel strategies to improve hearing restoration. Given the limitation of available treatments, we have observed a willingness among the hearing‐impaired to engage and try fundamental innovations in a collaborative ecosystem.

Current state‐of‐the‐art treatment modalities involve hearing aids and eCIs. eCIs present a strong benchmark as they enable speech understanding and have favorable safety and stability. Moreover, eCI continues to improve its usability, better or even full implantation and sound processing. Yet, these valuable developments do not target the real eCI bottleneck of limited spectral selectivity. Indeed, the eCI‐user perspective indicates a strong unmet clinical need. Preclinical data on oCIs suggest superior hearing restoration, but the feasibility, safety, and level of hearing improvement remain to be shown when bridging the species gap from rodents to non‐human primates, and finally to humans. Considering the requirement for gene therapy for optogenetic hearing restoration, the superiority in efficacy over the eCI needs to be convincing.

Thanks to advancements in basic research, curing genetic disorders by treating specific genes has become a reality. However, to translate gene therapy into the cochlea, further preclinical refinement is needed regarding high‐cell specificity, optimized vector delivery to the target site, and the controlled duration and extent of gene expression, while the therapy is administered within the therapeutic window and exerts no major adverse effects. In addition, regeneration of sensory cells or neurons represents a very promising strategy to restore hearing. Yet, risks and challenges regarding translation into humans are high and are likely to take decades: Cell regrowth needs to be confined, and replaced cells need to show target cell function and synaptically reconnect matching cochlear tonotopy to transmit auditory information.

Universally accessible broad genetic diagnostic testing for rapid identification of patients for gene therapy is important. However, even though gene therapy harbors the potential to restore a natural hearing perception in some cases, the translational efforts are tremendous and need to be redone for various genetic mutations for a limited number of patients benefiting from defective gene replacement therapies. Aiming to offer such specific, and thus expensive therapies to everyone, may require rethinking the society and insurance system.

Besides financial, ethical questions remain unclear: Does preclinical evidence for an improved new therapy mitigates translational risks to clinical trial participants? In return, is it ethically justified to withhold promising therapeutic approaches that could offer a greater quality of life? It remains to be defined at what threshold the preclinical evidence is sufficient for avoiding unnecessary risks and prolonged delays to maximize the quality of life. More support for academic research in the acceleration of therapy translation, and the possibility of involving and engaging patients at an early stage of therapy development need to be further developed.

### Information for patient questionnaire:

This study was approved through the “Ethik‐Kommission der Universität Göttingen” (Protocol # 23/11/19An). Questionnaires were distributed during routine check‐ups or by post, and were returned by post. Participation was voluntary and patients were informed that returned questionnaires implied consent.

**Pending issues**

i Proofing feasibility, safety, and level of hearing improvement by bridging the species gap from rodents to non‐human primates and humans.
ii Demonstrating superior efficacy of the oCI over the eCI in non‐human primates and humans.
iii Ensuring efficiency and biosafety of gene therapies, improvement of high‐cell specificity, vector delivery to the target site, and controlled duration and extent of gene expression.
iv Demonstrating long‐term biosafety and functional integration of transdifferentiated or transplanted hair cells or spiral ganglion neurons in patients.
v Can the health insurance system cover personalized medicine and how can we find an ethical balance of society‐financed and individual‐health aspects?
vi Support for academic research to accelerate translation and possibilities to involve patients at an early stage of therapy development to adapt to human needs.
vii Identify reliable ways of performing clinical trials on sophisticated novel therapies in efficient and reliable ways, even in small patient cohorts.

## Author contributions

**Bettina Julia Wolf:** Visualization. **Kathrin Kusch:** Visualization. **Victoria Hunniford:** Investigation; visualization. **Barbara Vona:** Validation. **Robert Kühler:** Investigation. **Daniel Keppeler:** Conceptualization; visualization; methodology. **Nicola Strenzke:** Conceptualization; resources. **Tobias Moser:** Conceptualization; resources; supervision; validation; visualization.

## Disclosure and competing interests statement

D.K. and T.M. are co‐founders of the OptoGenTech company that works towards clinical translation of the optical cochlear implant. T.M and N.S. hold grants to develop and improve diagnostics and discussed in this review and currently prepare an original manuscript on the questionnaire study with their collaborators.

## For more information

i https://www.omim.org/
ii https://www.orpha.net/
iii https://www.hearingloss.org/
iv http://www.auditory‐neuroscience.uni‐goettingen.de/hearing_the_light_EN.html
v https://www.innerearlab.uni‐goettingen.de/
vi https://www.cdc.gov/niosh/topics/noise/default.html
vii https://www.osha.gov/noise
